# Nationwide validation of the CLEO tool to evaluate the relevance of pharmacists’ interventions in German hospitals

**DOI:** 10.1007/s11096-025-02085-w

**Published:** 2026-02-17

**Authors:** Vivien Berger, Annika van der Linde, Lisa Cuba, Charlotte Horn, Denise Köster, Heike Lanzinger, Katharina Wien, Ha Thi Vo, Pierrick Bedouch, Claudia Langebrake

**Affiliations:** 1 Hospital Pharmacy, University Medical Center Hamburg-Eppendorf, Hamburg, Germany; 2 Pharmacy Department, Universitätsklinikum Erlangen and Friedrich-Alexander-Universität Erlangen-Nürnberg, Erlangen, Germany; 3 Pharmacy Department, Clinic Floridsdorf, Vienna Healthcare Group, Vienna, Austria; 4 Pharmacy Department, University Hospital Carl Gustav Carus, Dresden, Germany; 5 Institute of Medical Biometry and Epidemiology, University Medical Center Hamburg-Eppendorf, Hamburg, Germany; 6 Hospital Pharmacy, General Hospital Heidenheim, Heidenheim, Germany; 7 Hospital Pharmacy, University Hospital Schleswig-Holstein, Lübeck, Germany; 8 Pham Ngoc, Thach University of Medicine, Ho Chi Minh City, Vietnam; 9 Nguyen Tri Phuong Hospital, Ho Chi Minh City, Vietnam; 10 UF Pharmacie Clinique, Pôle Pharmacie, CHU Grenoble-Alpes, Grenoble, France; 11 TIMC, CNRS UMR5525, UFR de Pharmacie, University Grenoble-Alpes, Saint Martin d’Hères, France; 12 Department of Stem Cell Transplantation, University Medical Center Hamburg-Eppendorf, Hamburg, Germany

**Keywords:** Clinical pharmacy, Drug-related problems (DRPs), Medication error (ME), Medication review, Pharmacists’ intervention, Validation

## Abstract

**Introduction:**

The clinical relevance of pharmacists’ interventions (PIs) is complex to determine. The CLEO tool is a multidimensional scoring system to assess the relevance of PIs across three dimensions: clinical, economic, and organisational impact.

**Aim:**

This study aimed to nationwide validate the CLEO tool by clinical pharmacists in German hospitals using structured and representative clinical cases.

**Method:**

The German CLEO version was adapted to the German hospital setting and supplemented with practical examples. Fifty up-to-date cases from the inpatient setting were developed in a multistage process following the Identification, Situation, Background, Assessment, Recommendation structure. In the first round, each rater was randomly assigned 20 from the pool of 50 clinical cases, ensuring that all cases were evaluated by multiple raters. After a 14-day washout period, the same 20 cases were reassessed by the same raters. In the second round, all raters from the first round were invited again, and a subset who volunteered assessed another 20 cases after intensified training. Inter- and intra-rater reliability were calculated using Krippendorff’s α and the intraclass correlation coefficient (ICC). User feedback was collected through a 16-item questionnaire.

**Results:**

A total of 79 pharmacists from 56 hospitals participated in the first round; 27 completed the second round as well. Inter-rater reliability was poor across all three CLEO dimensions (Krippendorff’s α < 0.67), both overall and among experienced clinical pharmacists. Intra-rater reliability was good for all dimensions (ICC 0.63–0.74), highest for the clinical dimension (0.74). Most raters (77%) needed less than one minute per case. Overall, the CLEO tool was perceived by users as appropriate, precise, acceptable and feasible (mean score 5.36; 7-point Likert scale; 1 = strongly disagree, 7 = strongly agree).

**Conclusion:**

Since clinical pharmacy is still a developing discipline in German hospitals, differences in clinical practice and professional expertise complicate the evaluation of PIs. While intra-rater reliability was good, the validation of the CLEO tool in Germany did not achieve satisfactory inter-rater reliability. The CLEO tool may be useful within institutions with shared standards, but broader application across diverse settings in Germany requires additional training, further research and standardisation of clinical pharmacy services.

**Supplementary Information:**

The online version contains supplementary material available at https://doi.org/10.1007/s11096-025-02085-w.

## Impact statements

- We performed a nationwide validation of the CLEO tool in Germany using structured clinical cases based on the ISBAR framework, providing evidence to support the assessment of PIs for patient care.
- The validation demonstrates the existing heterogeneity in clinical pharmacy practice in Germany and emphasises the importance of targeted training and harmonised standards to support the future integration of validated tools like CLEO into clinical practice.
- We developed decision flowcharts as a practical tool to support the implementation of the CLEO tool in hospital practice.

## Introduction

Medication is the most common healthcare intervention to prevent, treat or manage diseases and health conditions [1]. Apart from the intended effect of a drug, patients may experience preventable and non-preventable harms. According to the Pharmaceutical Care Network Europe (PCNE), drug-related problems (DRPs) are defined as any event or circumstance involving drug therapy that actually or potentially interferes with desired health outcomes [2]. Besides negative effects on the efficacy and safety of the drug therapy, DRPs can also lead to high costs for the healthcare system [3–7]. As an overarching term, DRPs encompass several subgroups, including medication errors, adverse drug events, and adverse drug reactions [8, 9]. Among these, medication errors are of particular interest because addressing these errors could significantly reduce healthcare costs while improving patient safety. Different strategies have been proposed to minimise medication errors, including the involvement of clinical pharmacists [10–17]. Pharmacists’ interventions (PIs), which encompass a wide range of activities, aim to prevent and solve DRPs to optimise drug therapy [18–20].

While clinical pharmacy services have been well-established in many countries for years, Germany has not yet implemented nationwide standards in this area and continues to fall behind [21–23]. The extent and diversity of PIs in German hospitals are systematically documented within the framework of regularly conducted ‘intervention weeks’ [19]. During this one-month period, the German Association of Hospital Pharmacists (ADKA) invites hospital pharmacists to voluntarily document all PIs performed during a typical work week to generate nationwide benchmark data. To support the further development of clinical pharmacy services that enhance patient safety while reducing healthcare costs, it is essential to provide evidence of the benefits of PIs. Consequently, developing reliable methods to assess the relevance of PIs for patients is of great importance.

The National Coordinating Council for Medication Error Reporting and Prevention (NCC MERP) Index, the most widely used tool in clinical practice, in a first step categorises the severity of medication errors based on whether they reach the patient and subsequently according to the severity of the harm caused to the patient [24]. If medication errors are identified and solved early in the medication process before reaching the patient, the NCC MERP index may underestimate the relevance of PIs that prevent serious harm at an early stage. For example, data from the 2021 intervention week at the University Medical Center Hamburg-Eppendorf showed that 56.5% of PIs were classified as NCC MERP category B, which means that an error occurred but did not reach the patient [20].

Numerous alternative classification systems have been described in the literature. A systematic review by Vo et al. identified 82 tools for evaluating the potential impact of DRPs and/or PIs [25]. The evaluation of PIs proves to be complex, as they target different aspects of patient care, with some PIs primarily support the patient directly, while others also provide economic advantages or optimise organisational resources.

Since most existing classification systems focus primarily on the clinical impact, which refers to the anticipated effect of a PI on the patient’s clinical condition and overall health outcomes. Vo et al. developed a multidimensional classification system in France—the CLEO tool—which comprises three categories: clinical, economic and organisational [25–27]. The clinical dimension assesses the potential impact of an PI on patient outcomes from the patients’ perspective, the economic dimension considers hospital costs, and the organisational dimension evaluates effects on workflow, effort, and collaboration from the healthcare staff’s perspective. CLEO is designed to assess the relevance of overall PIs in the context of DRPs. A few years later, a German translation was developed in Switzerland, along with a validation that confirmed both effectiveness and user acceptance [28]. Recently, CLEO was successfully translated and validated in Vietnam [29]. However, previous validations often included only a small number of raters and/or cases that were not representative of the hospital setting in Germany.

### Aim

The aim of our study was to validate CLEO in the German hospital setting using up-to-date clinical cases from inpatient care presented in a structured format. We developed a low-threshold validation method with training materials and invited hospital pharmacists nationwide to assess the reliability of the CLEO tool for broad applicability.

## Method

### CLEO scale: Adaptation for the German hospital setting

The CLEO_de_ version from Switzerland, originally developed by Stämpfli et al., was adapted for the German hospital setting [28]. For this purpose, clinical pharmacists of the special interest group ‘pharmacists’ interventions’ hosted by the ADKA, participated in an iterative discussion process. While the structure of the three CLEO dimensions remained unchanged, the definitions were refined and supplemented with practical examples and flowcharts (see Results section for details). The “Not determined” category for each dimension of the CLEO scale was removed for the validation study.

### Clinical cases: From clinical practice to a structured format

Typical clinical cases from across various medical disciplines were collected by an expert clinical pharmacist at a University Medical Center, with an emphasis on actuality, hospital setting, and a structured format. The cases were developed using the ISBAR (Identification, Situation, Background, Assessment, Recommendation) framework, a structured handover concept for clinical practice to present patient cases systematically, including the introduction of the patient, the situation, relevant history, and the assessment, followed by a recommendation [30]. In a first step, 12 pilot cases were reviewed by five clinical pharmacists of the ADKA special interest group ‘pharmacists’ interventions’. Based on their feedback, the cases were refined and expanded to a final set of 50 cases, reflecting typical PIs reported from clinical practice. Although the cases were developed at a single center, an independent expert panel of five experienced clinical pharmacists from different German hospitals subsequently reviewed the 50 cases to ensure that they were perceived as representative and appropriate across multiple hospital settings. In an iterative online discussion each case was examined, and consensus was established using a majority agreement approach. To avoid bias, this second group of experts was excluded from the main validation.

### Validation process: Assessment of clinical pharmacists’ evaluations in two validation rounds

#### First validation round (V_1_)

**Fig. 1:**
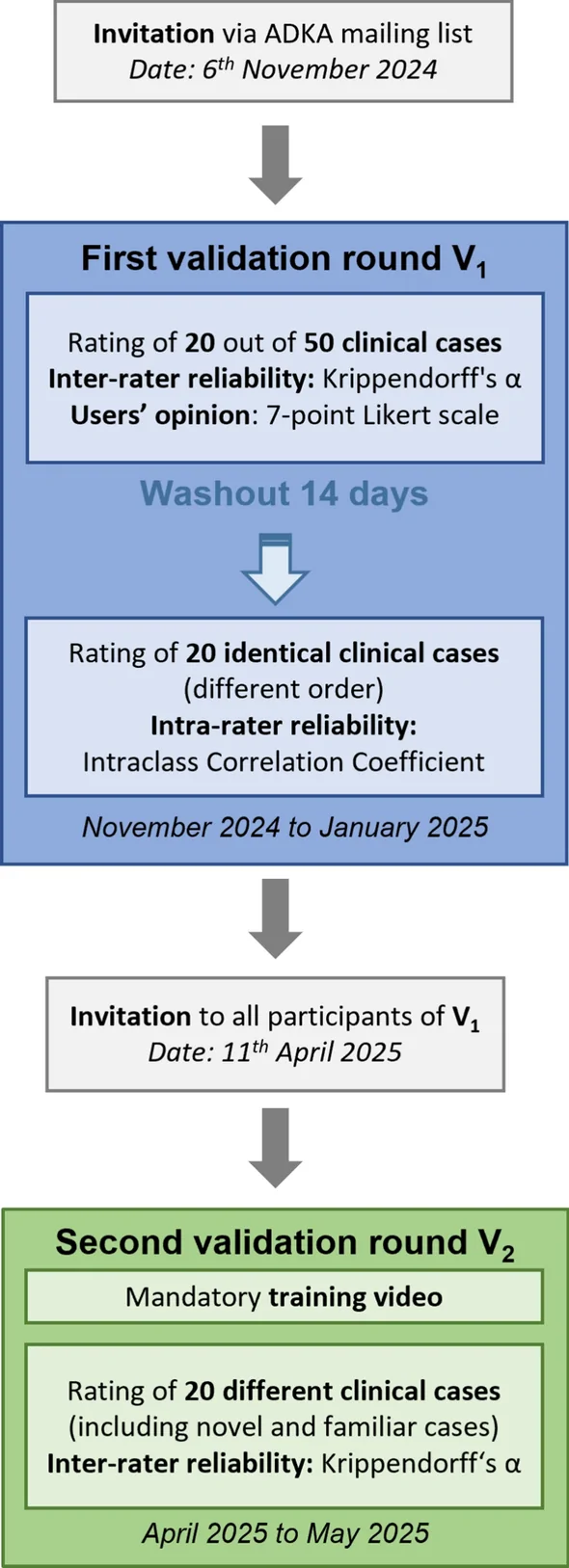
Validation process of the CLEO tool

Clinical pharmacists across Germany working in hospital settings were invited to participate in the CLEO validation via the ADKA mailing list on 6th November 2024, comprising 3,088 members (status November 2024). In the first validation round (V_1_), each rater was randomly assigned a set of 20 clinical cases from a total pool of 50 cases for evaluation across the three CLEO dimensions: clinical, economic and organisational. The randomisation was performed using R (version 4.5.0). After a 14-day washout period, each clinical pharmacist was asked to re-evaluate the same set of clinical cases presented in a different order. In V_1_, participants completed a self-study training based on the adapted CLEO scale with practical examples and flowcharts as training materials. The overall validation process is shown in Fig. 1.

#### Second validation round (V_2_)

In V_1_, substantial heterogeneity was observed in the ratings of the clinical cases, raising concerns about the adequacy of the initial training. Consequently, all participants from V_1_ were invited to voluntarily take part in a second round (V_2_) conducted five months later. In V_2_, this subset of participants evaluated the same sample of 20 clinical cases drawn from the full set of 50. Prior to the assessment, participants were provided with a video tutorial (PowerPoint presentation with audio) that included background information, case structure, and three sample ratings. All participants confirmed that they had watched the training video, and this was verified during the evaluation.

### Appropriateness, acceptability, precision, and feasibility: Evaluation of the CLEO tool itself

Apart from the clinical cases, the raters evaluated the CLEO tool in terms of appropriateness, acceptability, precision, and feasibility via an online questionnaire conducted in LimeSurvey (https://www.limesurvey.org/de). The questionnaire contained a total of 16 questions, based on a modified version by AbuRuz [31], which was previously used in other studies [26, 28, 29]. The agreement on the four dimensions was assessed using a 7-point Likert scale (1 = strongly disagree, 7 = strongly agree), with results visualised in Excel (Microsoft Corporation, Redmond, United States, version 2016). The survey was conducted within V_1_.

### Statistics: Evaluation of inter- and intra-rater reliability

All statistical analyses were conducted using SPSS Statistics (IBM, Armonk, United States, version 29). Inter-rater reliability was assessed using Krippendorff’s α for ordinal data involving more than two raters, which also accounts for the number of possible categories [32]. We integrated a macro developed by Hayes and Krippendorff in SPSS [33]. A value of α ≥ 0.80 indicates high agreement and reliability, 0.67 to 0.79 moderate, and below 0.67 poor inter-rater reliability [34, 35]. We calculated the intra-rater reliability using the intraclass correlation coefficient (ICC), specifically a two-way mixed-effects model with single measurements and absolute agreement (ICC_A,1_) and then determined the means ($\overline{{{\mathrm{ICC}}}}_{{{\mathrm{A}},{1}}}$) for each dimension. According to Cicchetti, we interpreted the $\overline{{{\mathrm{ICC}}}}_{{{\mathrm{A}},{1}}}$: < 0.40 as poor, 0.40 to 0.59 as fair, 0.60 to 0.74 as good, and 0.75 to 1.0 as excellent [36].

### Ethics approval

In accordance with German regulations on research involving human participants, this study did not require ethical approval, since no patient-identifiable information was accessed [37].

## Results

As a first step, the CLEO scale was adapted and expanded to address the needs of hospital settings in Germany. The adaptation primarily concerned the clinical dimension, whose original definitions refer to the prevention of harm or the avoidance of prolonged hospital stays. The limitation of this definition is that some PIs do not directly prevent harm. For example, clinical pharmacists may recommend initiating a statin in a patient with elevated cardiovascular risk, which supports long-term evidence-based therapy even if it does not avert immediate harm. Therefore, the definitions within the clinical dimension were expanded to include PIs that contribute to an appropriate drug therapy. Another important adjustment concerned the addition of typical practical examples to the scale, such as avoiding under- or overdoses in long-term drug therapy, failure to adjust doses in cases of organ dysfunction, avoiding duplicate prescriptions and avoiding drug-drug interactions (supplementary file 1). The clinical relevance of these examples may vary depending on the drug, patient and clinical setting, with potential impacts ranging from minor clinical relevance to major or even life-threatening consequences. Along with the practical examples, decision flowcharts were developed to guide raters through each dimension of the CLEO scale (supplementary file 2).

A total of 50 clinical cases were developed based on the ISBAR framework. Table 1 provides an overview of three selected cases from the validation, along with the corresponding reason for a PI (according to ADKA DokuPIK classification [38]) and the CLEO ratings.

**Table 1:** Clinical case examples following the ISBAR framework, reason for PI and associated CLEO ratings

*Example 1:*	
**I**dentification	**I**: 72-year-old woman
**S**ituation	**S**: Medical speciality: Cardiology. Current diagnosis: Pyelonephritis
**B**ackground	**B**: Empiric treatment of pyelonephritis started four days ago with IV ceftriaxone (2 g once daily). Enterococcus faecalis was identified in the urine culture.
**A**ssessment	**A**: Empiric therapy with ceftriaxone is being continued, despite the availability of a targeted therapy option based on the antibiogram. Ceftriaxone also increases the risk of a Clostridioides difficile infection.
**R**ecommendation	**R**: Targeted antibiotic therapy for E. faecalis detection with IV ampicillin (2 g three times daily). The pathogen is tested as sensitive in the antibiogram. The costs for both antibiotic regimens are comparable.
**Reason for PI**	Inappropriate or not most suitable drug in terms of indication
CLEO rating	
***Cl****inical*	**Moderate (2)** → The PI contributes to adequate and/or guideline-based drug therapy.
**E**conomic	**No change (0)** → The PI does not lead to any change in the hospital's direct costs.
**O**rganisational	**Negative (-1)** → The PI has a negative impact on the time and effort required for the treatment process.
*Example 2:*	
**I**dentification	**I**: 84-year-old man
**S**ituation	**S**: Medical speciality: ENT (Ear, nose and throat)
**B**ackground	**B**: Medical history: Rheumatoid arthritis treated with methotrexate (MTX) 15 mg orally once weekly. Renal function: eGFR = 63 ml/min
**A**ssessment	**A**: Medication error: On admission to the ward, MTX was incorrectly prescribed at a dose of 15 mg orally once daily. The patient has been taking this incorrect dose for the last five days.
**R**ecommendation	**R**: Adjustment of the dosing interval to once weekly. Supportive measures (IV hydration, calcium folinate) will be carried out under hematological monitoring on the normal ward. Daily intake of MTX may result in fatal toxicity.
**Reason for PI**	(Inappropriate) dose
*CLEO rating*	
**Cl**inical	**Avoids fatality (4)** → The PI prevents serious to potentially life-threatening harm.
**E**conomic	**Increase in cost (-1)** → The PI increases the hospital's direct costs due to additional monitoring (pharmacological measures and blood-level monitoring).
**O**rganisational	**Positive (1)** → The PI improves the level of knowledge within the treatment process.
*Example 3:*	
**I**dentification	**I**: 56-year-old woman
**S**ituation	**S**: Medical speciality: Gynecology
**B**ackground	**B**: Long-term medication: Citalopram tablets 20 mg, taken once daily in the evening (indication: depression). Clinical notes: The patient reports sleep disorders during inpatient stay.
**A**ssessment	**A**: Citalopram has a stimulating effect and is usually taken in the morning.
**R**ecommendation	**R**: Change the administration time of citalopram from evening to morning.
**Reason for PI**	Administration (duration)
*CLEO rating*	
**Cl**inical	**Minor (1)** → The PI has a low clinical impact on the patient in terms of knowledge and quality of life.
**E**conomic	**No change (0)** → The PI does not lead to any change in the hospital's direct costs.
**O**rganisational	**Neutral (0)** → The PI has no impact on the treatment process.

As part of V_1_, a total of 79 raters from 56 different hospital settings took part. Approximately one third of the initial participants (n = 27), took part in V_2_ of the validation process. Table 2 provides a comprehensive overview of the participant characteristics. All types of hospitals were represented, with a particular focus on University hospitals. Two-thirds of the raters regularly participate in interdisciplinary ward rounds (without direct patient contact).

**Table 2:** Characteristics of participants in V_1_ and V_2_

	V_1_		V_2_	
	Number	Per cent	Number	Per cent
*Type of hospital*				
University hospital	30	38.0	10	37.1
Maximum care hospital	19	24.0	8	29.6
Hospital of full medical care, specialist hospital	8	10.1	3	11.1
General hospital	21	26.6	6	22.2
Other	1	1.3	0	0
*Pharmaceutical focus of daily work (multiple selection possible)*				
Ward rounds (without patient contact)	50	63.3	13	48.1
Drug information service	41	51.9	8	29.6
Ward rounds (with patient contact)	29	36.7	11	40.7
Logistics and medication dispensing	26	32.9	8	29.6
Consultation service	19	24.1	14	51.9
Medication review supported by a CPOE/CDSS^a^	19	24.1	8	29.6
Medication reconciliation at hospital admission	13	16.5	4	14.8
Pharmaceutical manufacturing (non-sterile compounding, cytotoxic preparation)	7	8.9	2	7.4
Discharge management	2	2.5	0	0
*Professional experience of participating pharmacists*				
Expert clinical pharmacist (according to advanced training)	47	59.5	20	74.1
*Clinical pharmacy services, years*				
< Five years	28	35.4	8	29.6
Five to ten years	24	30.4	10	37.1
> Ten years	27	34.2	9	33.3
**Total participants**	**79**	**100.0**	**27**	**100.0**

^a^*CPOE/CDSS* Computerised Physician Order Entry / Clinical Decision Support System

### Inter-rater reliability

In V_1_, a total of 79 raters participated, and the results are summarised in Table 3. The inter-rater reliability values for all three dimensions, calculated using Krippendorff’s α, remained below 0.67 for all dimensions, resulting in a poor level of agreement. Among a subgroup of 31 raters of V_1_ with at least ten years of professional experience in clinical pharmacy services, inter-rater reliability was consistently lower across all three dimensions compared to the overall sample in V_1_. In V_2_, 27 raters participated. While the agreement improved for the clinical dimension, the economic and organisational dimensions showed lower inter-rater reliability compared to V_1_. Across all dimensions, inter-rater reliability remained below 0.67.

### Intra-rater reliability

Following a 14-day washout period, 77% of the initial raters (61 out of 79) completed the reassessment required to calculate intra-rater reliability. All three dimensions demonstrated intra-rater reliability values ($\overline{{{\mathrm{ICC}}}}_{{{\mathrm{A}},{1}}}$) greater than 0.60, indicating good reliability. The clinical dimension showed the highest intra-rater reliability with a $\overline{{{\mathrm{ICC}}}}_{{{\mathrm{A}},{1}}}$ of 0.74.

**Table 3:** Summary of inter- and intra-rater reliability results from the validation process

Validation round	Rater (n)	Type of reliability	CLEO categories		
			Clinical	Economic	Organisational
**V**_**1**_ (full sample)	79	**Inter-rater**	0.48	0.55	0.35
**V**_**1**_ (subgroup ≥ ten years)	31	**Inter-rater**	0.42	0.49	0.31
**V**_**2**_ (subsample from V_1_)	27	**Inter-rater**	0.54	0.47	0.31
**V**_**1**_ (14-day washout)	61	**Intra-rater**	0.74	0.69	0.63

### User feedback

**Fig. 2:**
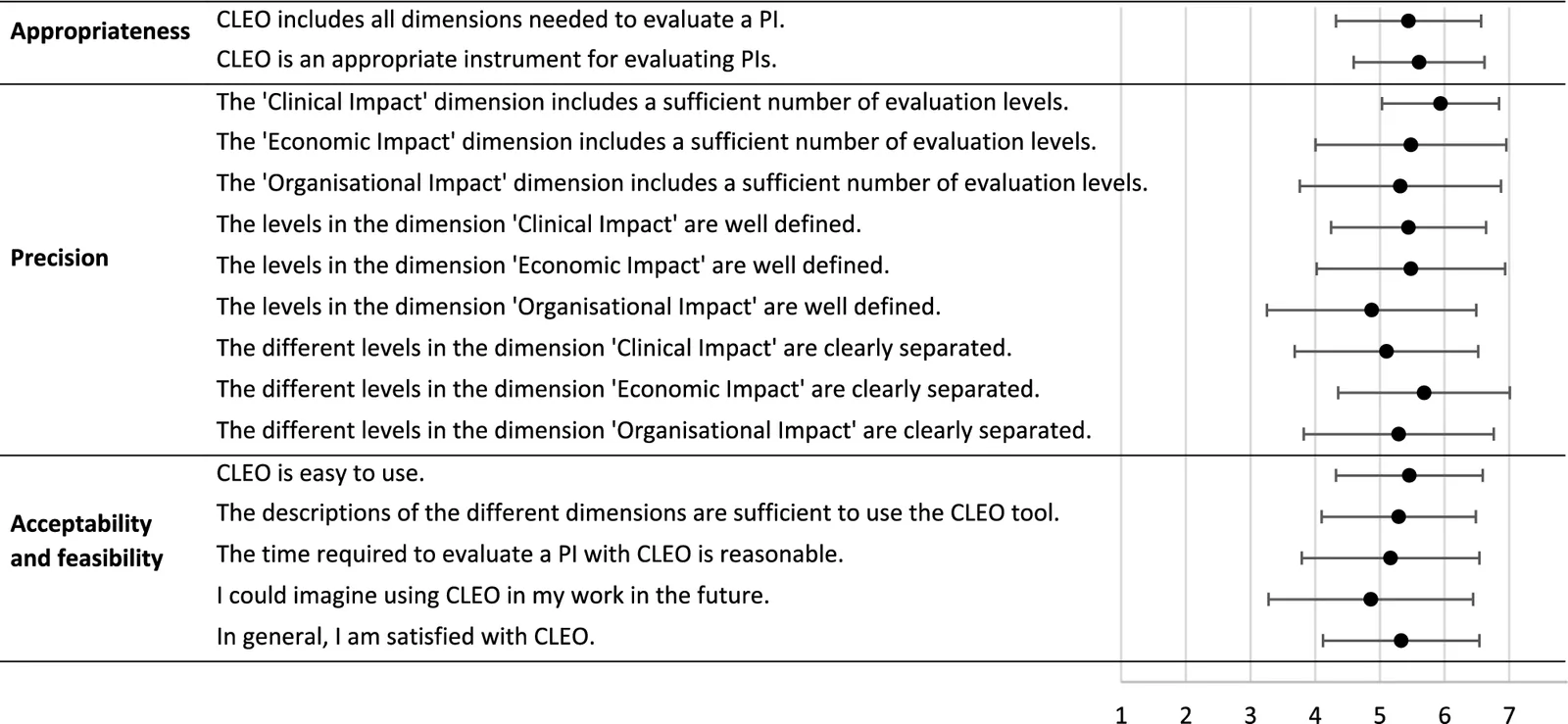
Users’ agreement on appropriateness, precision, acceptability and feasibility of the CLEO tool was measured using a 7-point Likert scale (1 = strongly disagree, 4 = neutral, 7 = strongly agree; mean ± standard deviation)

The full sample of V_1_, comprising a total of 79 raters, responded to a total of 16 questions regarding the CLEO tool, its individual dimensions, and its applicability in clinical practice. The mean user agreement rating was 5.36 on the 7-point Likert scale. A detailed overview of the results is provided in Fig. 2. CLEO was perceived as appropriate (mean = 5.53; standard deviation = 1.07), precise (5.40; 1.38) as well as acceptable and feasible (5.22; 1.30) for evaluating the potential relevance of PIs. The average time required by raters to assess one clinical case varied, with 23% needing more than 1 min, 58% between 30 s and 1 min, and 19% less than 30 s.

## Discussion

We adapted the CLEO tool to the context of clinical pharmacy practice in German hospitals, introducing content modifications and supplementing the original scale with practical examples. The adapted version was validated in a nationwide study involving hospital pharmacists from all levels of care and with diverse professional experience, thus reflecting the heterogeneity of clinical practice in German hospitals. Intra-rater reliability was consistently good across all dimensions, whereas inter-rater reliability remained at a poor level. Overall, raters evaluated the adapted CLEO tool as a suitable and well-accepted instrument for assessing the relevance of PIs.

### Factors influencing reliability

Several factors can influence the reliability estimates of validation studies and should be considered when interpreting the results. Beyond the scoring tool itself, important variables include the characteristics of the clinical cases, the study objective, the number, qualifications, and training of the raters, as well as the statistical approach applied. Contextual aspects such as institutional practices and cultural differences may also play a role. The following sections discuss these factors in more detail. Table 4 summarises validation characteristics, methods, and outcomes across the different CLEO studies.

**Table 4:** Comparison of CLEO studies

Study objective	Original development of CLEO [39]	German translation and validation [28]	Vietnamese translation and validation [29]	German adaptation and nationwide validation
Author(s), published year	Vo et al., 2015	Stämpfli et al., 2019	Nguyen et al,. 2024	Berger et al., (present study)
Country, setting	France Six hospitals	Switzerland Three hospitals	Vietnam Six hospitals	Germany 56 hospitals
Number, structure and source of clinical cases	30 for internal and 60 cases for external reliability Descriptive cases Retrospective from database	Ten cases Descriptive cases Literature and CLEO validation study	100 cases Descriptive cases Retrospective from nine hospitals	20 out of 50 cases Structured case format Newly designed cases
Quality and number of raters	Internal: seven experts of the French Society of Clinical Pharmacy External: seven expert clinical pharmacists	Ten clinical pharmacists Different level of experience	Seven clinical pharmacists Minimum five years of experience	Inter-rater: 79 (V_1_) *vs*. 27 (V_2_) Intra-rater: 61 clinical pharmacists Different level of experience
Rating method	Individual-based rating	Individual-based rating	Individual-based rating	Individual-based rating
Inter-rater reliability	Intra-class correlation (internal *vs.* external)	Intra-class correlation	Intra-class correlation	Krippendorff’s α (V_1_ *vs.* V_2_)
CL	0.69 *vs.* 0.65	0.63	0.71	0.48 *vs.* 0.54
E	0.82 *vs.* 0.81	0.65	0.86	0.55 *vs.* 0.47
O	0.42 *vs.* 0.50	0.30	0.56	0.35 *vs.* 0.31
Intra-rater reliability	Intra-class correlation Washout: one month	Intra-class correlation Washout: seven days	Intra-class correlation Washout: one month	Intra-class correlation Washout: 14 days
CL	0.82	0.76	0.79	0.74
E	0.92	0.85	0.84	0.69
O	0.74	0.53	0.56	0.63

*CL* clinical, *E* economic, *O* organisational, *V*_1_ first validation round, *V*_2_ second validation round

In the original study by Vo et al., the “Not determined” option was not provided to raters during the validation. This option was added after validation of the study. Similarly, in the validation study conducted in Vietnam, this category was not available. Therefore, we also omitted this category for the purpose of validation to ensure that raters would not select it prematurely without thoroughly evaluating the available information. Only in the Swiss validation by Stämpfli et al. this option was directly provided to raters.

### Structure of clinical cases

A literature review showed that most model cases in validation studies were derived from databases or validated cases from the literature [28, 29, 39–42]. Unlike the typical descriptive format, we used a structured approach to present information in an easily accessible way and to minimise raters’ perception of being tested [43, 44]. To achieve this, we used the ISBAR framework for clinical handovers [45]. The time required for evaluation was generally short with 77% of raters indicating that assessing the three CLEO dimensions took less than one minute.

### Study objective

Differences in reliability outcomes across studies may in part be explained by the different study objectives. While previous studies primarily aimed to validate the original CLEO tool within small expert panels, the present study focused on adapting the scale to German hospital practice and testing its reliability at a national level, with the aim of integrating it into the national ADKA-DokuPIK database. This is particularly relevant as the number and selection of raters can substantially influence the outcomes of validation studies [46]. Compared to previous CLEO validations, which included only seven to ten raters from three to six hospitals, our study involved a substantially larger number of raters (27 to 79) and more than 50 different hospitals [26, 28, 29, 40, 42]. We invited all interested hospital pharmacists in Germany to participate, irrespective of their professional or scientific experience. By selecting a small group of experts from a limited number of sites, the results may yield more favourable reliability estimates, as their perspectives on clinical practice are likely more aligned, leading to more consistent assessments. In terms of methodology, it is recommended to increase the number of raters to enhance the validity and generalisability of the results [47–49]. Thus, the high number of involved raters may partly explain the substantial variability in our findings.

The heterogeneity observed in the ratings during V_1_ indicates that raters may have been evaluating the underlying DRP and its severity rather than the PI, as it was actually intended. To address this potential training-related problem, a mandatory training video—including case examples to explain the classification into the three dimensions of the CLEO tool—was implemented prior to V_2_. Nevertheless, the training intervention had no measurable effect on inter-rater reliability.

### Differences in statistical approaches

Before implementation in clinical practice, scoring systems must be evaluated in terms of reliability and validity, as these represent the most essential standardised criteria for any scientific measurement instrument [50–52]. The reliability estimates evaluate the stability of measures, including inter-rater reliability (agreement between different raters) and intra-rater reliability (consistency of repeated assessments). In our study, inter-rater reliability was calculated using Krippendorff’s α, a method suitable for multiple raters, ordinal data, and datasets with missing values [33]. Compared with CLEO validations in France, Switzerland, and Vietnam [28, 29, 39] inter-rater reliability in our study was substantially lower. This difference may partly be due to the use of Krippendorff’s α, a more conservative method that generally yields lower values than the ICC used in previous CLEO validations [53–55]. Intra-rater reliability was assessed via a test–retest approach, for which we calculated the ICC after a 14-day washout period, consistent with the methodology applied in previous CLEO studies [28, 29, 39].

### Contextual factors in German hospitals

Beyond methodological differences, contextual factors may also account for the observed discrepancies. In France, the CLEO tool has been well-established for many years and is considered the gold standard, as reflected by its use in multiple national studies across both inpatient and outpatient settings [56–58]. The higher reliability values reported in the Swiss study may likewise reflect contextual advantages, as the CLEO tool had already been integrated into hospital pharmacy practice before evaluation. In addition, the number of cases and raters was more limited in Switzerland. Moreover, although both validations were conducted in German, the CLEO version applied in Switzerland was not identical to our adapted version. These methodological differences may at least partly explain the higher reliability values observed in the Swiss study.

Another factor that may have contributed to the heterogeneous results in our German cohort is the variability in the scope and intensity of clinical pharmacy services across institutions [23]. Currently, there are no national standards for ward-based clinical pharmacy services in Germany, and practice levels differ across institutions. This local variation likely contributes to inconsistencies in the evaluation of PIs. The ADKA is in the process of developing a national standard to address this issue.

### Implications for clinical practice

The validation followed methodological standards to enable wider implementation of the CLEO tool in Germany. While this broader approach may naturally lead to greater variability in ratings, it provides a more realistic view of the tool’s applicability in routine practice. Nevertheless, the results did not demonstrate sufficient reliability, irrespective of the raters’ experience or the intensity of the training provided. However, the use of the CLEO tool can still be valuable if applied under standardised conditions by trained raters. Importantly, it provides a validated, easy-to-use method to document the value of clinical pharmacy services, which remains highly relevant for healthcare policy and for demonstrating the impact of PIs. Moreover, the CLEO tool has been increasingly adopted internationally, illustrating its growing acceptance across different healthcare systems [59–61]. Nevertheless, our findings emphasise that a validation status of a scoring system alone does not guarantee applicability across diverse clinical contexts. The specific conditions under which a tool was validated must be critically evaluated prior to the integration into daily practice.

### Strengths and limitations

One of the strengths of our validation approach was the large number of participating clinical pharmacists and the diversity of the involved hospital settings. There is a potential risk of selection bias, as individuals with a higher level of motivation may have been more likely to participate. A novel aspect of our validation design was the use of the ISBAR structure. Due to the concise structure, raters may have received insufficient information or found certain cases difficult to interpret. The inclusion of the same raters in both validation rounds may cause learning effects. A general limitation is that clinical pharmacists assess the relevance of their own work.

## Conclusion

The nationwide validation of the CLEO tool in Germany demonstrated that the number and selection of the raters, but also the diversity of hospital settings have a considerable influence on the outcomes. Despite training intervention, inter-rater reliability across all three dimensions for the CLEO tool remained poor. These findings strongly indicate the need for further research to develop a broadly applicable and reliable approach for assessing the relevance of PIs. Nevertheless, CLEO may still be useful in smaller, well-aligned groups operating under shared standards and defined frameworks.

## Supplementary Information

Below is the link to the electronic supplementary material.

Supplementary file1 (PDF 204 KB)

Supplementary file2 (PDF 196 KB)

## Data Availability

The clinical cases and datasets from this study are available from the corresponding author upon reasonable request.

## References

[1] World Health Organization. Medication safety in polypharmacy: a WHO technical report. 2019.

[2] Pharmaceutical Care Network Europe (PCNE). PCNE Classification for Drug-Related Problems V9.1. 2020.Available from: https://www.pcne.org/upload/files/417_PCNE_classification_V9-1_final.pdf.

[3] van den Bemt PM, Egberts TC, de Jong-van den Berg LT, et al. Drug-related problems in hospitalised patients. Drug Saf. 2000;22(4):321–33. 10.2165/00002018-200022040-00005. 10789826

[4] Pirmohamed M, Breckenridge AM, Kitteringham NR, et al. Adverse drug reactions. BMJ. 1998;316(7140):1295–8. 10.1136/bmj.316.7140.1295. PMC1113033 9554902

[5] Silva C, Ramalho C, Luz I, et al. Drug-related problems in institutionalized, polymedicated elderly patients: opportunities for pharmacist intervention. Int J Clin Pharm. 2015;37(2):327–34. 10.1007/s11096-014-0063-2. 25637404

[6] Gautier S, Bachelet H, Bordet R, et al. The cost of adverse drug reactions. Expert Opin Pharmacother. 2003;4(3):319–26. 10.1517/14656566.4.3.319. 12614184

[7] Rottenkolber D, Hasford J, Stausberg J. Costs of adverse drug events in German hospitals: a microcosting study. Value Health. 2012;15(6):868–75. 10.1016/j.jval.2012.05.007. 22999137

[8] Linden-Lahti C, Takala A, Holmstrom AR, et al. Applicability of drug-related problem (DRP) classification system for classifying severe medication errors. BMC Health Serv Res. 2023;23(1):743. 10.1186/s12913-023-09763-3. PMC10334531 37430249

[9] Aly AF. Definitionen zu Pharmakovigilanz und Arzneimitteltherapiesicherheit (AMTS) (Definitions of pharmacovigilance and drug therapy safety). Arzneiverordnung Prax. 2015;42:99–104. 10.1016/j.zefq.2012.10.023 23217722

[10] Benjamin DM. Reducing medication errors and increasing patient safety: case studies in clinical pharmacology. J Clin Pharmacol. 2003;43(7):768–83. 10.1177/0091270003254794 12856392

[11] Reis CT, Paiva SG, Sousa P. The patient safety culture: a systematic review by characteristics of Hospital Survey on Patient Safety Culture dimensions. Int J Qual Health Care. 2018;30(9):660–77. 10.1093/intqhc/mzy080. 29788273

[12] Hodkinson A, Tyler N, Ashcroft DM, et al. Preventable medication harm across health care settings: a systematic review and meta-analysis. BMC Med. 2020;18(1):313. 10.1186/s12916-020-01774-9. PMC7646069 33153451

[13] Durand M, Castelli C, Roux-Marson C, et al. Evaluating the costs of adverse drug events in hospitalized patients: a systematic review. Health Econ Rev. 2024;14(1):11. 10.1186/s13561-024-00481-y. PMC10851489 38329561

[14] Bates DW, Spell N, Cullen DJ, et al. The costs of adverse drug events in hospitalized patients. Adverse Drug Events Prevention Study Group. JAMA. 1997;277(4):307–11. 10.1001/jama.1997.03540280045032. 9002493

[15] Bond CA, Raehl CL, Franke T. Clinical pharmacy services, pharmacy staffing, and the total cost of care in United States hospitals. Pharmacotherapy. 2000;20(6):609–21. 10.1592/phco.20.7.609.35169. 10853615

[16] Kaboli PJ, Hoth AB, McClimon BJ, et al. Clinical pharmacists and inpatient medical care: a systematic review. Arch Intern Med. 2006;166(9):955–64. 10.1001/archinte.166.9.955. 16682568

[17] Viktil KK, Blix HS. The impact of clinical pharmacists on drug-related problems and clinical outcomes. Basic Clin Pharmacol Toxicol. 2008;102(3):275–80. 10.1111/j.1742-7843.2007.00206.x. 18248511

[18] ADKA DokuPIK. Dokumentation Pharmazeutischer Interventionen im Krankenhaus (Documentation of pharmacists' interventions in the hospital). 2025.https://www.adka-dokupik.de/. Accessed 11 Aug 2025.

[19] Langebrake C, Hohmann C, Lezius S, et al. Clinical pharmacists’ interventions across German hospitals: results from a repetitive cross-sectional study. Int J Clin Pharm. 2022;44(1):64–71. 10.1007/s11096-021-01313-3. PMC8866273 34402022

[20] Berger V, Sommer C, Boje P, et al. The impact of pharmacists’ interventions within the Closed Loop Medication Management process on medication safety: an analysis in a German university hospital. Front Pharmacol. 2022;13:1030406. 10.3389/fphar.2022.1030406. PMC9704051 36452222

[21] Ludewig T, Schoeffski O, Langebrake C. Die klinisch-pharmazeutische Visite – eine Bestandsaufnahme (The clinical-pharmaceutical ward round – a survey). Krankenhauspharmazie. 2018;39:467–74.

[22] Frontini R, Miharija-Gala T, Sykora J. EAHP survey 2010 on hospital pharmacy in Europe: parts 4 and 5. Clinical services and patient safety. Eur J Hosp Pharm. 2013;20(2):69–73. 10.1136/ejhpharm-2013-000285.

[23] Schulz C, Fischer A, Vogt W, et al. Clinical pharmacy services in Germany: a national survey. Eur J Hosp Pharm. 2021;28(6):301–5. 10.1136/ejhpharm-2019-001973. PMC8552150 34697045

[24] NCC MERP. National Coordinating Council on Medication Error Reporting and Prevention. NCC MERP Index for Categorizing Medication Errors. 2001.https://www.nccmerp.org/types-medication-errors. Accessed 20 Aug 2025.

[25] Vo TH, Charpiat B, Catoire C, et al. Tools for assessing potential significance of pharmacist interventions: a systematic review. Drug Saf. 2016;39(2):131–46. 10.1007/s40264-015-0370-0. 26650064

[26] Vo HT, Charpiat B, Chanoine S, et al. CLEO: a multidimensional tool to assess clinical, economic and organisational impacts of pharmacists’ interventions. Eur J Hosp Pharm. 2021;28(4):193–200. 10.1136/ejhpharm-2020-002642. PMC8239266 33883205

[27] Vo HT. Evaluation of the potential impact of pharmacist interventions: development and validation of the CLEO multidimensional tool (Doctoral dissertation, Université Grenoble Alpes). Université Grenoble Alpes; 2015.

[28] Staempfli D, Baumgartner P, Boeni F, et al. Translation and validation of a tool to assess the impact of clinical pharmacists’ interventions. Int J Clin Pharm. 2019;41(1):56–64. 10.1007/s11096-018-0755-0. 30478493

[29] Nguyen ATT, Nguyen KHP, Le HB, et al. Translation and validation of the CLEO tool in Vietnamese to assess the significance of pharmacist interventions. Int J Clin Pharm. 2024. 10.1007/s11096-024-01813-y. 39365523

[30] Burgess A, van Diggele C, Roberts C, et al. Teaching clinical handover with ISBAR. BMC Med Educ. 2020;20(Suppl 2):459. 10.1186/s12909-020-02285-0. PMC7712559 33272274

[31] AbuRuz SM, Bulatova NR, Yousef AM. Validation of a comprehensive classification tool for treatment-related problems. Pharm World Sci. 2006;28(4):222–32. 10.1007/s11096-006-9048-0. 17066238

[32] Zapf A, Castell S, Morawietz L, et al. Measuring inter-rater reliability for nominal data - which coefficients and confidence intervals are appropriate? BMC Med Res Methodol. 2016. 10.1186/s12874-016-0200-9. PMC4974794 27495131

[33] Hayes AF, Krippendorff K. Answering the call for a standard reliability measure for coding data. Commun Methods Meas. 2007;1(1):77–89. 10.1080/19312450709336664.

[34] Krippendorff K. Reliability in content analysis - some common misconceptions and recommendations. Hum Commun Res. 2004;30(3):411–33. 10.1093/hcr/30.3.411.

[35] Krippendorff K. Content analysis: an introduction to its methodology: Sage publications; 2018. ISBN: 9781071878781.

[36] Cicchetti DV. Guidelines, criteria, and rules of thumb for evaluating normed and standardized assessment instruments in psychology. Psychol Assess. 1994;6(4):284.

[37] Aerztekammer Hamburg. Berufsordnung der Hamburger Aerzte und Aerztinnen vom 27.03.2000 i.d.F. vom 06.09.2021 (Professional code of conduct for Hamburg physicians dated March 27, 2000, as amended on September 6, 2021).https://aerztekammer-hamburg.org/wp-content/uploads/2025/01/Berufsordnung-gueltig-ab1-03-2022_CD.pdf. Accessed 11 Aug 2023.

[38] Ihbe-Heffinger A, Langebrake C, Hohmann C, et al. Prospective survey-based study on the categorization quality of hospital pharmacists’ interventions using DokuPIK. Int J Clin Pharm. 2019;41(2):414–23. 10.1007/s11096-019-00785-8. 30895502

[39] Vo TH, Charpiat B, Juste M, et al. Development of a multidimensional scale “CLEO” for evaluation of clinical, economic, and organizational impacts of a pharmacist intervention. Int J Clin Pharm. 2015;37(1):184–5.

[40] Overhage JM, Lukes A. Practical, reliable, comprehensive method for characterizing pharmacists’ clinical activities. Am J Health Syst Pharm. 1999;56(23):2444–50. 10.1093/ajhp/56.23.2444. 10595804

[41] Ganso M, Areschin S, Lange P, et al. Verlaesslichkeit eines Klassifikationssystems fuer pharmazeutische Interventionen (Reliability of a classification system for pharmacists’ interventions). Krankenhauspharmazie. 2007;28(7):273.

[42] Maes KA, Tremp RM, Gsasa Working group on clinical pharmacy, et al. Demonstrating the clinical pharmacist's activity: validation of an intervention oriented classification system. Int J Clin Pharm. 2015;37(6):1162–71. 10.1007/s11096-015-0179-z. 26290379

[43] Miall DS. Anticipation and feeling in literary response, a neuropsychological perspective. Poetics. 1995;23(4):275–98. 10.1016/0304-422x(95)00004-4.

[44] Lovis C, Baud RH, Planche P. Power of expression in the electronic patient record: structured data or narrative text? Int J Med Inform. 2000;58–59:101–10. 10.1016/s1386-5056(00)00079-4. 10978913

[45] Marshall S, Harrison J, Flanagan B. The teaching of a structured tool improves the clarity and content of interprofessional clinical communication. Qual Saf Health Care. 2009;18(2):137–40. 10.1136/qshc.2007.025247. 19342529

[46] Kottner J, Audigé L, Brorson S, et al. Guidelines for reporting reliability and agreement studies (GRRAS) were proposed. J Clin Epidemiol. 2011;64(1):96–106. 10.1016/j.jclinepi.2010.03.002. 21130355

[47] Gwet KL. Handbook of inter-rater reliability: the definitive guide to measuring the extent of agreement among raters: Advanced Analytics, LLC; 2014. ISBN: 9780970806284.

[48] Shrout PE, Fleiss JL. Intraclass correlations: uses in assessing rater reliability. Psychol Bull. 1979;86(2):420–8. 10.1037/0033-2909.86.2.420. 18839484

[49] Fleiss JL, Levin B, Paik MC. Statistical methods for rates and proportions. Wiley; 2013.

[50] Kimberlin CL, Winterstein AG. Validity and reliability of measurement instruments used in research. Am J Health Syst Pharm. 2008;65(23):2276–84. 10.2146/ajhp070364. 19020196

[51] DeVon HA, Block ME, Moyle-Wright P, et al. A psychometric toolbox for testing validity and reliability. J Nurs Scholarsh. 2007;39(2):155–64. 10.1111/j.1547-5069.2007.00161.x. 17535316

[52] Scholtes VA, Terwee CB, Poolman RW. What makes a measurement instrument valid and reliable? Injury. 2011;42(3):236–40. 10.1016/j.injury.2010.11.042. 21145544

[53] Sertdemir Y, Burgut HR, Alparslan ZN, et al. Comparing the methods of measuring multi-rater agreement on an ordinal rating scale: a simulation study with an application to real data. J Appl Stat. 2013;40(7):1506–19. 10.1080/02664763.2013.788617.

[54] Hallgren KA. Computing inter-rater reliability for observational data: an overview and tutorial. Tut Quant Methods Psychol. 2012;8(1):23–34. 10.20982/tqmp.08.1.p023. PMC3402032 22833776

[55] Koo TK, Li MY. A guideline of selecting and reporting intraclass correlation coefficients for reliability research. J Chiropr Med. 2016;15(2):155–63. 10.1016/j.jcm.2016.02.012. PMC4913118 27330520

[56] Arcani V, Protzenko D, Henri J, et al. Assessing the impact of pharmacist-led interventions in an academic pain clinic: clinical outcomes and interprofessional insights. Eur J Pain. 2025. 10.1002/ejp.70062. PMC12202836 40572026

[57] Duwez M, Chanoine S, Lepelley M, et al. Clinical evaluation of pharmacists’ interventions on multidisciplinary lung transplant outpatients’ management: results of a 7-year observational study. BMJ Open. 2020;10(11):e041563. 10.1136/bmjopen-2020-041563. PMC7703423 33247028

[58] Novais T, Maldonado F, Grail M, et al. Clinical, economic, and organizational impact of pharmacists’ interventions in a cognitive-behavioral unit in France. Int J Clin Pharm. 2021;43(3):613–20. 10.1007/s11096-020-01172-4. 33052482

[59] Silva S, Jesus M, Faria S, et al. Impact of pharmacist interventions in a Portuguese hospital: a study using the CLEO multidimensional tool. Pharmacy. 2025. 10.3390/pharmacy13050143. PMC12567203 41149871

[60] Reinau D, Furrer C, Stämpfli D, et al. Evaluation of drug-related problems and subsequent clinical pharmacists’ interventions at a Swiss university hospital. J Clin Pharm Ther. 2019;44(6):924–31. 10.1111/jcpt.13017. 31408206

[61] Sudeshika T, Deeks LS, Naunton M, et al. Evaluating the potential outcomes of pharmacist-led activities in the Australian general practice setting: a prospective observational study. Int J Clin Pharm. 2023;45(4):980–8. 10.1007/s11096-023-01604-x. PMC10239215 37269443

