# Supplementary material for: Nationwide validation of the CLEO tool to evaluate the relevance of pharmacists’ interventions in German hospitals
Source: Int J Clin Pharm. 2026 Feb 17;48(3):897–908. doi: 10.1007/s11096-025-02085-w (PMC13176046; doi:10.1007/s11096-025-02085-w)
Supplement: Supplementary file 1 — Supplementary file1 (PDF 204 KB) [file 11096_2025_2085_MOESM1_ESM.pdf]

Nationwide validation of the CLEO tool to evaluate the relevance of pharmacists' interventions in German hospitals

– Supplementary file 1 –

Vivien Berger[1], Annika van der Linde[1], Lisa Cuba[2,3], Charlotte Horn[4], Denise Köster[5], Heike Lanzinger[6], Katharina Wien[7], Ha Thi Vo[8,9], Pierrick Bedouch[10,11], Claudia Langebrake[1,12]

[1]Hospital Pharmacy, University Medical Center Hamburg-Eppendorf, Hamburg, Germany

[2]Pharmacy Department, Universitätsklinikum Erlangen and Friedrich-Alexander-Universität Erlangen-Nürnberg, Erlangen, Germany

[3]Pharmacy Department, Clinic Floridsdorf, Vienna Healthcare Group, Vienna, Austria

[4]Pharmacy Department, University Hospital Carl Gustav Carus, Dresden, Germany

[5]Institute of Medical Biometry and Epidemiology, University Medical Center Hamburg-Eppendorf, Hamburg, Germany

[6]Hospital Pharmacy, General Hospital Heidenheim, Heidenheim, Germany

[7]Hospital Pharmacy, University Hospital Schleswig-Holstein, Lübeck, Germany

[8]Pham Ngoc Thach University of Medicine, Ho Chi Minh City, Vietnam

[9]Nguyen Tri Phuong Hospital, Ho Chi Minh City, Vietnam

[10]UF Pharmacie clinique, Pôle Pharmacie, CHU Grenoble-Alpes, Grenoble, France

[11]TIMC, CNRS UMR5525, UFR de Pharmacie, University Grenoble-Alpes, Saint Martin d'Hères, France

[12]Department of Stem Cell Transplantation, University Medical Center Hamburg-Eppendorf, Hamburg, Germany

International Journal of Clinical Pharmacy

Corresponding Author:
Vivien Berger
Hospital Pharmacy, University Medical Center Hamburg-Eppendorf, Hamburg, Germany,
Martinistraße 52, 20246 Hamburg, Germany. v.berger@uke.de

Disclaimer:
This English version is a direct translation of the German adapted $CLEO_{de}$ scale. It has not been validated and may contain errors in wording, grammar, sentence structure, or meaning.

Supplementary file 1. Evaluation of the impact of pharmacists' interventions (PIs) by the adapted CLEO$_{de}$ scale

Clinical Impact		
**Principle:**	The clinical impact is assessed <u>based on the most probable scenario</u> rather than the worst/best scenario.<br>The clinical impact is evaluated from the <u>patient's perspective</u>.	
**Explanation:**	**Harm**	Physical harm, impairment of the patient's mental and/or physical abilities, and/or resulting pain. The term 'harm' also includes inadequate drug therapy in relation to compliance with current treatment guidelines.
	**Quality of life**	Physical aspects (autonomy, physical abilities, capacity to perform tasks of daily life, etc.).<br>Psychological aspects (anxiety, depression, emotionality, etc.).<br>Social aspects (relative to family environment, friends or professional contacts, engaging in personal relationships, participation in social and leisure activities etc.)<br>Somatic aspects (symptoms of the disease).
	**Monitoring**	Follow-up checks (blood pressure, heart rate, respiratory rate, level of consciousness, etc.), and laboratory monitoring.
	**Treatment**	Change of therapy or an additional medical/surgical treatment.

**Impact**	**Definition CLEO$_{de}$** (adapted)	**Practical examples**
**Harmful/ negative (-1)**	The pharmacists' intervention (PI) may lead to **negative outcomes** regarding the patient's clinical condition, level of knowledge, satisfaction, adherence, and/or quality of life.	• Wrong/negative recommendation due to disregard of important patient- or treatment-related aspects
**Unchanged (0)**	The PI has **no impact** on the patient regarding clinical condition, level of knowledge, satisfaction, adherence, and/or quality of life.	• Prevention of errors through general advice or information that is not patient-specific<br>• General information, e. g.: administration of drugs via feeding tubes, dosage/dosing interval, pharmaeconomic, drug shortages and alternatives<br>• Patient-related pharmacoeconomic PIs<br>• Switching to formulary drugs (aut-simile, aut-idem)
**Minor (1)**	The PI has a **minor impact** on the patient regarding knowledge, satisfaction, adherence and/or quality of life.	• Avoidance of duplicate prescriptions<br>• Adjustment of the dosing interval and/or dosage form<br>• Discontinuation/pausing of a drugs<br>• Optimisation of administration times (exception: drug-drug interactions)<br>} … with **minor clinical impact**<br>• Counseling/training of the patient regarding drug administration, side effects and administering information<br>• Increase of adherence through prescription of combination drugs

**Moderate (2)**	The PI has a **high clinical impact** or **prevents moderate harm**<br>*OR*<br>contributes to **adequate** and/or **guideline-compliant drug therapy**.	• Avoidance of duplicate prescriptions<br>• Avoidance of under- or overdosing of drugs in long-term therapy<br>• Adjustment of the dosing interval and/or dosage form<br>• Missing dose adjustment in impaired organ function<br>• Adjustment of drugs for gastric or jejunal feeding tubes<br>• Discontinuation/pausing of drugs<br>} … with **high clinical impact**<br>• **Therapy optimisation based on**:<br>– Appropriate/relevant therapeutic standards (guidelines, local standards, protocols, or literature)<br>– Prescription of recommended drugs to prevent side effects<br>– Switching from parenteral to oral therapy with equivalent efficacy<br>– Implementation/compliance with therapeutic drug monitoring for drugs without potential organ toxicity<br>– De-escalation of calculated anti-infective therapy when the pathogen is known (and susceptibility testing if available)
**Major (3)**	The PI **prevents potentially toxic or serious harm** to the patient.	• Adjustment of dosage, dosing interval, dosage form, or route of administration<br>• Substantial overdose of drugs with a wide therapeutic range<br>• Substantial underdosing<br>• Missing dose adjustment in impaired organ function<br>• Too low/too high dosing of drugs for acute conditions<br>• Drug-drug interactions or duplicate prescriptions<br>• Discontinuation/pausing of drugs<br>• Prescription of an incorrect drug (e. g., mix-up, sound-alike)<br>} … Prevention of **potentially toxic or serious consequences**<br>• Prescription of a drug that may worsen the patient's condition (e.g., ignoring a relative contraindication or a known intolerance)<br>• Implementation/compliance with therapeutic drug monitoring for substances that can cause potential organ toxicity<br>• Failure to prescribe necessary drugs to prevent organ damage or serious consequences
**Vital (4)**	The PI **prevents severe to life-threatening harm** to the patient (e. g. intensive care treatment).	• Substantial overdose of drugs with a narrow therapeutic range<br>• Failure to prescribe or underdosing of potentially life-saving drugs<br>• Severe drug-drug interactions<br>• Implementation/compliance with therapeutic drug monitoring<br>} … Prevention of **potentially life-threatening consequences**<br>• Discontinuation/pausing of drugs

Economic Impact	
**Principle:**	The economic impact refers to the direct costs for the hospital (treatment costs). Medical and non-medical direct costs are all a consumption of resources resulting from a disease and its treatment.
**Explanation:**	The **treatment costs** include the following aspects:<br>• Drug costs<br>• Costs for monitoring drug therapy (e.g., follow-up checks, laboratory tests, therapeutic drug monitoring, etc.)<br>• Diagnostic examinations

Impact	Definition CLEO$_{de}$ (adapted	Practical examples
**Higher costs (-1)**	The PI **increases** the hospital's direct costs.	• Prescription of additional budget-relevant drugs<br>• Additional laboratory tests or therapeutic drug monitoring<br>• Switching to alternative drugs/individual imports in case of drug shortages<br>• Shortening of the dosing interval
**Null (0)**	The PI **does not change** the hospital's direct costs.	• Change of administration times<br>• Information on drug selection, dosage, administration, etc.<br>• Information on ZE/NUB[1] status<br>• Pausing/discontinuation/prescription of drugs (exception: budget-relevant drugs)
**Lower costs (1)**	The PI **reduces** the hospital's direct costs.	• Pausing/discontinuation of budget-relevant drugs<br>• Switching to formulary drugs

[1] ZE/NUB: Additional reimbursement for new diagnostic or treatment methods under the German DRG system

Organisational Impact	
**Principle:**	The organisational impact takes into account the workload, workflow and/or collaboration in the treatment process from the perspective of the medical staff.
**Explanation:**	The following **aspects** should particularly be taken into consideration:<br>• Time saving<br>• facilitating tasks or teamwork<br>• Improved knowledge/information sharing<br>• improved security for staff<br>• Simplification of professional tasks<br>• Continuity of care

Impact	Definition CLEO$_{de}$ (adapted)	Practical examples
**Worsened (-1)**	The PI has a **negative effect** on the workload, workflow, and/or collaboration within the course of treatment.	• More frequent administration of a drug (e.g., shortening of the dosing interval)<br>• Switch from oral to intravenous drug formulation<br>• Increased time consumption for procurement/preparation/administration of drugs<br>• Additional laboratory tests or therapeutic drug monitoring<br>• Prolonged infusion time of intravenous drugs
**Null (0)**	The PI has **no impact** on the workflow, workload, or collaboration in the treatment process.	• Therapy adjustments without influence on the interval or dosage form (e. g. change of drug or dose)<br>• Change of administration times<br>• Switching to formulary drugs or changes due to drug shortages<br>• Prescription/pausing/discontinuation of drugs
**Improved (1)**	The PI **improves** the workflow, increases knowledge, or reduces workload within the treatment process.	• Training/counselling of nursing staff<br>• Information on the correct use of drugs<br>• Information on administering drugs via enteral feeding tubes<br>• Oralisation of intravenous drugs<br>• Workload reduction through preparation of drugs in the hospital pharmacy<br>• Staff safety through preparation of CMR[2] substances in the hospital pharmacy

[2] CMR: Carcinogenic, mutagenic, reprotoxic
