# Supplementary material for: Nationwide validation of the CLEO tool to evaluate the relevance of pharmacists’ interventions in German hospitals
Source: Int J Clin Pharm. 2026 Feb 17;48(3):897–908. doi: 10.1007/s11096-025-02085-w (PMC13176046; doi:10.1007/s11096-025-02085-w)
Supplement: Supplementary file 2 — Supplementary file2 (PDF 196 KB) [file 11096_2025_2085_MOESM2_ESM.pdf]

Nationwide validation of the CLEO tool to evaluate the relevance of pharmacists' interventions in German hospitals

– Supplementary file 2 –

Vivien Berger[1], Annika van der Linde[1], Lisa Cuba[2,3], Charlotte Horn[4], Denise Köster[5], Heike Lanzinger[6], Katharina Wien[7], Ha Thi Vo[8,9], Pierrick Bedouch[10,11], Claudia Langebrake[1,12]

[1]Hospital Pharmacy, University Medical Center Hamburg-Eppendorf, Hamburg, Germany

[2]Pharmacy Department, Universitätsklinikum Erlangen and Friedrich-Alexander-Universität Erlangen-Nürnberg, Erlangen, Germany

[3]Pharmacy Department, Clinic Floridsdorf, Vienna Healthcare Group, Vienna, Austria

[4]Pharmacy Department, University Hospital Carl Gustav Carus, Dresden, Germany

[5]Institute of Medical Biometry and Epidemiology, University Medical Center Hamburg-Eppendorf, Hamburg, Germany

[6]Hospital Pharmacy, General Hospital Heidenheim, Heidenheim, Germany

[7]Hospital Pharmacy, University Hospital Schleswig-Holstein, Lübeck, Germany

[8]Pham Ngoc Thach University of Medicine, Ho Chi Minh City, Vietnam

[9]Nguyen Tri Phuong Hospital, Ho Chi Minh City, Vietnam

[10]UF Pharmacie clinique, Pôle Pharmacie, CHU Grenoble-Alpes, Grenoble, France

[11]TIMC, CNRS UMR5525, UFR de Pharmacie, University Grenoble-Alpes, Saint Martin d'Hères, France

[12]Department of Stem Cell Transplantation, University Medical Center Hamburg-Eppendorf, Hamburg, Germany

International Journal of Clinical Pharmacy

Corresponding Author:

Vivien Berger

Hospital Pharmacy, University Medical Center Hamburg-Eppendorf, Hamburg, Germany, Martinistraße 52,

20246 Hamburg, Germany. v.berger@uke.de

Disclaimer:

This English version is a direct translation of the German flowcharts. It has not been validated and may contain errors in wording, grammar, sentence structure, or meaning.

Supplementary material 2. Flowcharts for the evaluation of pharmacists' interventions using the adapted $CLEO_{de}$ scale

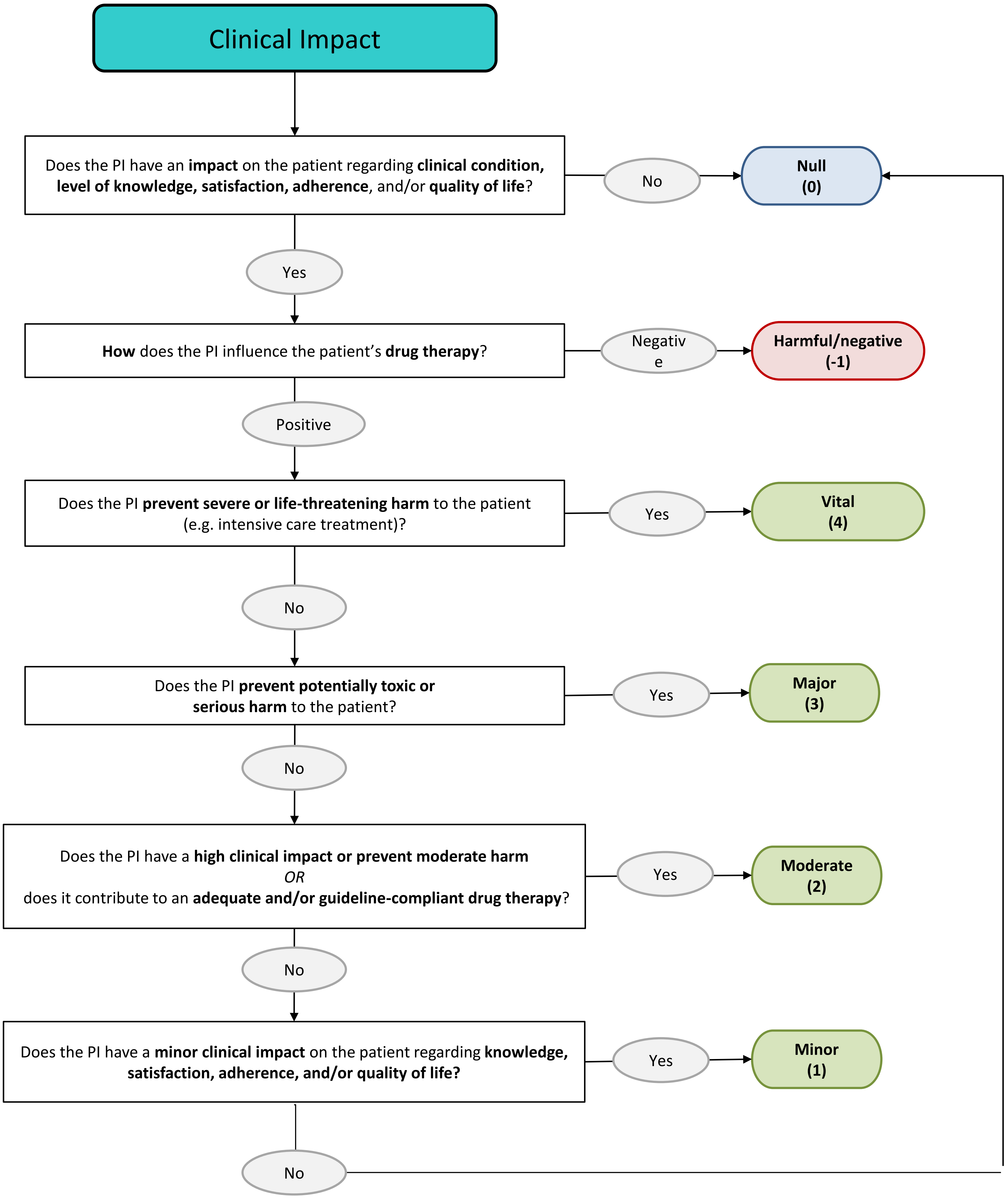

**Principle:**	The clinical impact is assessed based on the most probable scenario rather than the worst/best scenario.<br>The clinical impact is evaluated from the patient's perspective.	
**Explanation:**	**Harm**	Physical harm, impairment of the patient's mental and/or physical abilities, and/or resulting pain.<br>The term 'harm' also includes inadequate drug therapy in relation to compliance with current treatment guidelines.
	**Quality of life**	• Physical aspects (autonomy, physical abilities, capacity to perform tasks of daily life, etc.).<br>• Psychological aspects (anxiety, depression, emotionality, etc.).<br>• Social aspects (relative to family environment, friends or professional contacts, engaging in personal relationships, participation in social and leisure activities, etc.).<br>• Somatic aspects (symptoms of the disease).
	**Monitoring**	Follow-up checks (blood pressure, heart rate, respiratory rate, level of consciousness, etc.), and laboratory monitoring.
	**Treatment**	Change of therapy or additional medical/surgical treatment.

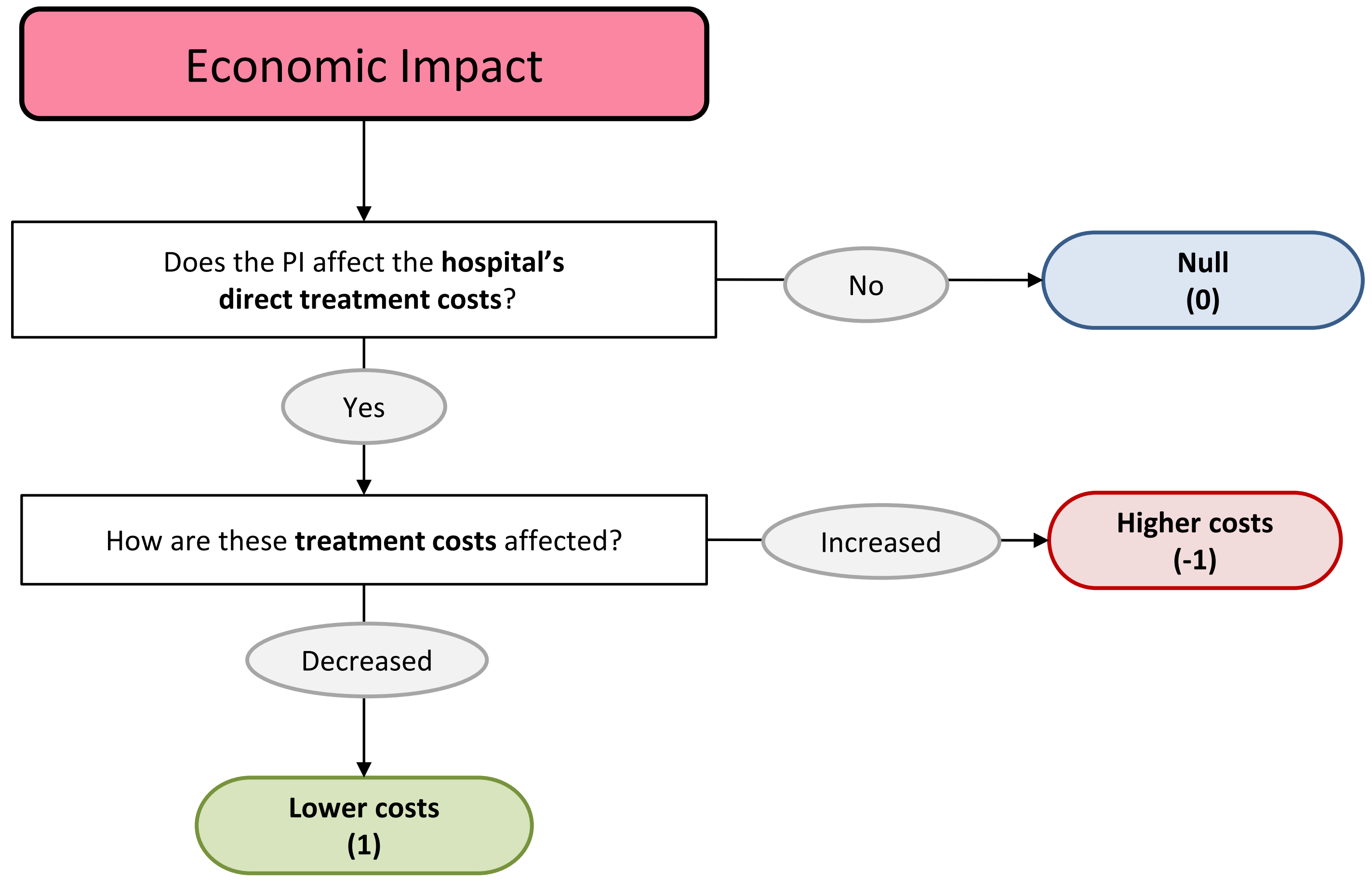

**Principle:**	The economic impact refers to the <u>direct costs of the hospital (treatment costs)</u>. Medical and non-medical direct costs are all resource consumptions resulting from a disease and its treatment.
**Explanation:**	The treatment costs include the following aspects:<br>• Drug costs<br>• Costs for monitoring drug therapy (e.g., follow-up controls, laboratory tests, therapeutic drug monitoring, etc.)<br>• Diagnostic examinations

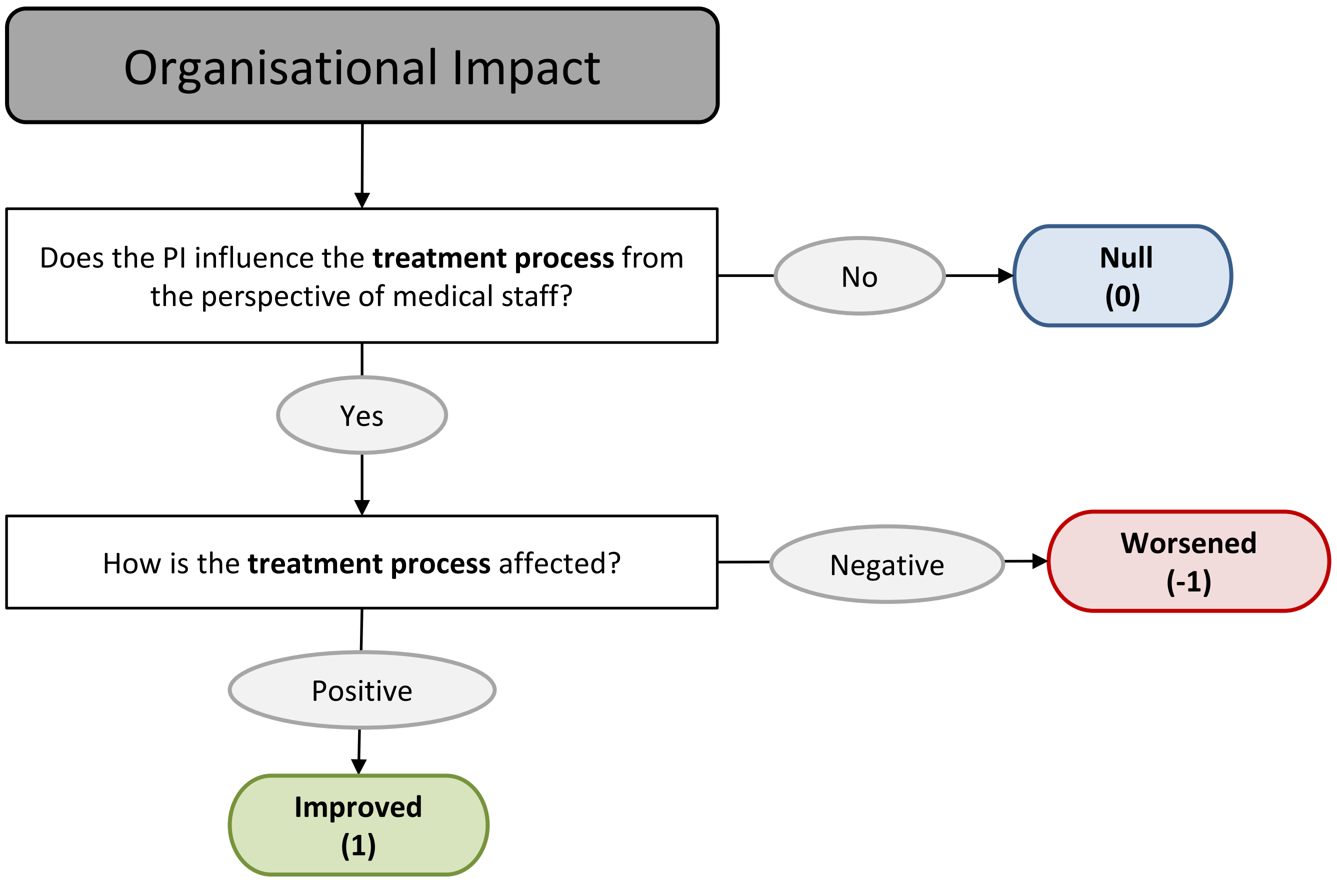

**Principle:**	The organisational impact takes into account the <u>workload, workflow and/or collaboration in the treatment process from the perspective of medical staff.</u>	
**Explanation:**	The following **aspects** should be particularly considered:	
	• Time saving	• Facilitating tasks or teamwork
	• Improved knowledge/information sharing	• Inproved security for staff
	• Simplification of professional tasks	• Continuity of care
